# Surveillance for sulfadoxine-pyrimethamine resistant malaria parasites in the Lake and Southern Zones, Tanzania, using pooling and next-generation sequencing

**DOI:** 10.1186/s12936-017-1886-9

**Published:** 2017-06-05

**Authors:** Jeremiah M. Ngondi, Deus S. Ishengoma, Stephanie M. Doctor, Kyaw L. Thwai, Corinna Keeler, Sigsbert Mkude, Oresto M. Munishi, Ritha A. Willilo, Shabbir Lalji, Naomi Kaspar, Chonge Kitojo, Lynn A. Paxton, Nicholas J. Hathaway, Jeffrey A. Bailey, Jonathan J. Juliano, Steven R. Meshnick, Julie Gutman

**Affiliations:** 1 RTI International, Dar es Salaam, Tanzania; 2 National Institute for Medical Research, Tanga, Tanzania; 3 UNC Gillings School of Global Public Health, Chapel Hill, NC USA; 4 National Malaria Control Programme, Dar es Salaam, Tanzania; 5 US President’s Malaria Initiative/United States Agency for International Development, Dar es Salaam, Tanzania; 6 US President’s Malaria Initiative, Malaria Branch, Division of Parasitic Diseases and Malaria, US Centers for Disease Control and Prevention, Dar es Salaam, Tanzania; 7 Program in Bioinformatics and Integrative Biology, University of Massachusetts Medical School, Worcester, MA USA; 8 Malaria Branch, Division of Parasitic Diseases and Malaria, US Centers for Disease Control and Prevention, Atlanta, GA USA

**Keywords:** *Plasmodium falciparum*, Sulfadoxine-pyrimethamine, Tanzania, Malaria, Resistance

## Abstract

**Background:**

Malaria in pregnancy (MiP) remains a major public health challenge in areas of high malaria transmission. Intermittent preventive treatment in pregnancy (IPTp) with sulfadoxine-pyrimethamine (SP) is recommended to prevent the adverse consequences of MiP. The effectiveness of SP for IPTp may be reduced in areas where the *dhps*581 mutation (a key marker of high level SP resistance) is found; this mutation was previously reported to be common in the Tanga Region of northern Tanzania, but there are limited data from other areas. The frequency of molecular markers of SP resistance was investigated in malaria parasites from febrile patients at health centres (HC) in seven regions comprising the Lake and Southern Zones of mainland Tanzania as part of the ongoing efforts to generate national-wide data of SP resistance.

**Methods:**

A cross-sectional survey was conducted in the outpatient departments of 14 HCs in seven regions from April to June, 2015. 1750 dried blood spot (DBS) samples were collected (117 to 160 per facility) from consenting patients with positive rapid diagnostic tests for malaria, and no recent (within past 2 months) exposure to SP or related drugs. DNA was extracted from the DBS, pooled by HC, and underwent pooled targeted amplicon deep sequencing to yield estimates of mutated parasite allele frequency at each locus of interest.

**Results:**

The *dhps*540 mutation was common across all 14 sites, ranging from 55 to 98.4% of sequences obtained. Frequency of the *dhps*581 mutation ranged from 0 to 2.4%, except at Kayanga HC (Kagera Region, Lake Zone) where 24.9% of sequences obtained were mutated. The *dhfr*164 mutation was detected only at Kanyanga HC (0.06%).

**Conclusion:**

By pooling DNA extracts, the allele frequency of mutations in 14 sites could be directly determined on a single deep-sequencing run. The *dhps*540 mutant was very common at all locations. Surprisingly, the *dhps*581 was common at one health center, but rare in all the others, suggesting that there is geographic micro-heterogeneity in mutant distribution and that accurate surveillance requires inclusion of multiple sites. A better understanding of the effect of the *dhps*581 mutant on the efficacy of IPTp-SP is needed.

## Background

Malaria in pregnancy (MiP) remains a major public health challenge in areas of high malaria transmission. In pregnant women, malaria can cause mild to severe maternal anaemia, particularly in primigravid women, and placental infection can interfere with the maternal-fetal exchange of nutrients and oxygen, leading to preterm delivery and low birth weight, and consequently increasing neonatal mortality [1, 2]. In order to prevent the adverse consequences of MiP, the World Health Organization (WHO) recommends the use of intermittent preventative treatment in pregnancy (IPTp) with sulfadoxine-pyrimethamine (SP)—a full treatment dose administered to pregnant women during routine ANC visits in the 2nd and 3rd trimesters. IPTp has a protective efficacy of nearly 25% against low birth weight and 21% against neonatal mortality [3].

*Plasmodium falciparum* resistance to SP results from an ordered accumulation of mutations in two genes, namely *P. falciparum dihydrofolate reductase* (*Pfdhfr*) and *P. falciparum dihydropteroate synthase* (*Pfdhps*) that code for enzymes targeted by sulfadoxine and pyrimethamine, respectively. Resistance increases with the number of mutant alleles. The presence of five mutant alleles, the *dhfr/dhps* “quintuple mutant”, including the *dhfr* substitutions N51**I**, C59**R**, and S108**N** and the *dhps* substitutions A437**G** and K540**E**, are associated with a very high rate of failure when SP is used for the treatment of uncomplicated falciparum malaria [4]. However, SP remains effective for IPTp, even where the prevalence of the quintuple mutant is high, and thus its use continues to be recommended by the WHO [5, 6]. The efficacy of SP for IPTp appears to be compromised in the presence of a sixth mutation at *dhps*581 (sextuple mutant; A581**G**); infection with parasites harbouring the sextuple mutant has been associated with increased placental parasitaemia and inflammation and failure of IPTp-SP to improve birth weight [7–9].

Previous studies have reported that the *dhps* 581 mutation is common in Tanga Region of Tanzania, with up to 57% of parasites harbouring the sextuple mutant [10]; however, there are limited data from other areas of Tanzania. In light of the possibility that IPTp-SP may provide no benefit to women in areas with a high prevalence of the sextuple mutant, it is critical to define the extent of this mutation. This study aimed to investigate the prevalence of the SP resistance mutations *dhps* K540**E** (a surrogate marker for quintuple mutant [4]) and *dhps* A581**G** [9] and *dhfr* I164**L** [1] (surrogate markers for sextuple mutant parasites, with A581**G** being the more widely reported) in the parasite population in the Lake Zone (Mwanza, Geita, Mara, and Kagera Regions) and Southern Zone (Lindi, Mtwara, and Ruvuma Regions) of Tanzania.

## Methods

### Study area

**Fig. 1:**
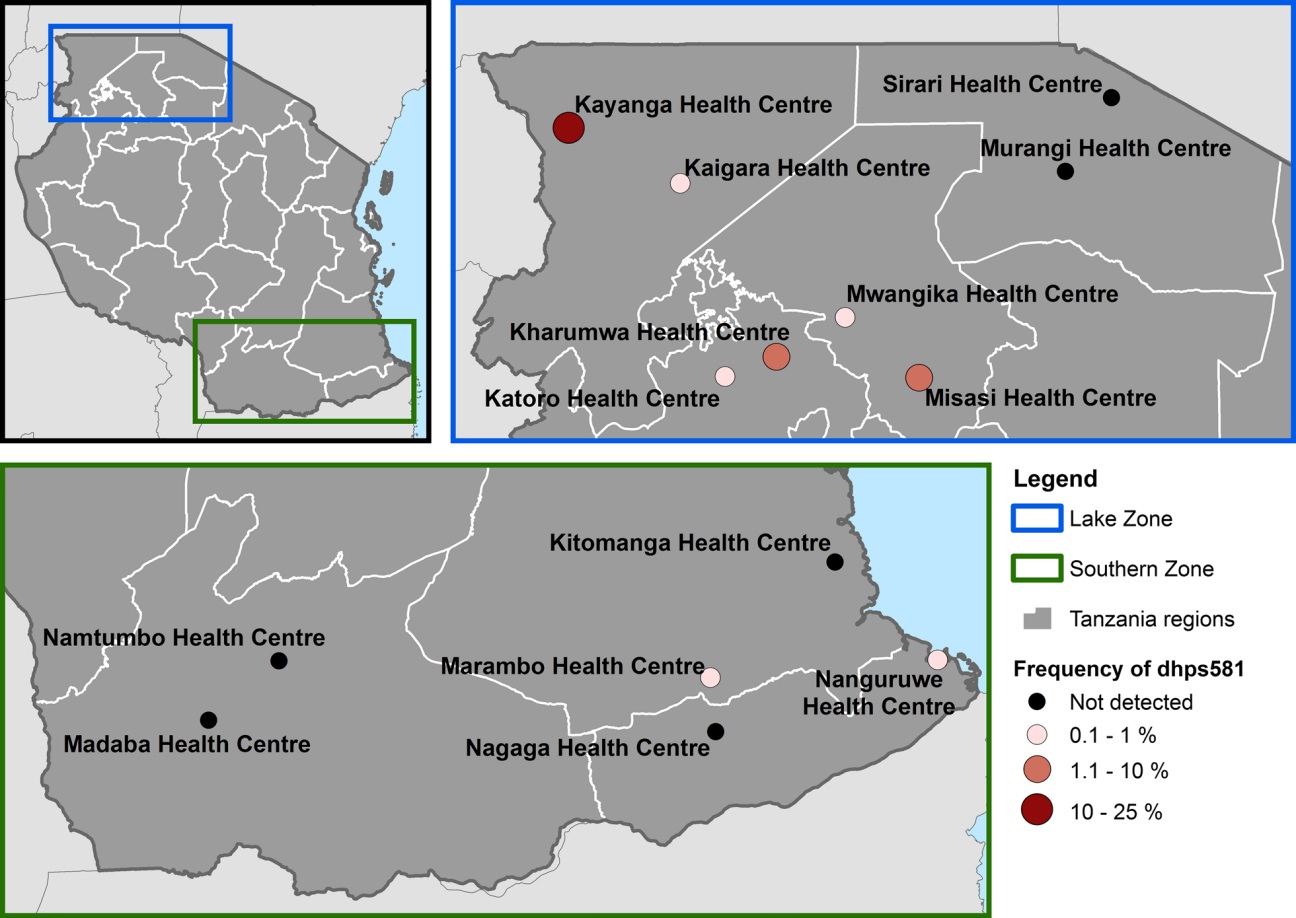
Location of sampled health facilities with frequency of *dhps*A581**G** mutations

From April to June, 2015, a cross-sectional survey was conducted in 14 health facilities (HFs) in seven regions of mainland Tanzania: all four regions in the Lake Zone (Mwanza, Geita, Mara, and Kagera) and all three regions in the Southern Zone (Lindi, Mtwara, and Ruvuma Regions) (Fig. 1). These regions were selected as they had high prevalence of malaria in the 2011/12 Tanzania HIV and Malaria Indicator Survey (THMIS) [11]. In each region, two health facilities were conveniently sampled from facilities with laboratory and where staff had been previously trained on collection of malaria rapid diagnostic tests (RDTs) for other projects to determine whether the *dhps*A581**G** mutation was present in these regions.

### Sample size

It was estimated that a sample size of 100 malaria positive patients from each facility would produce a two-sided 95% confidence interval of 1.6–11.3%, assuming the true proportion of patients infected with a given mutation was 5%. To account for false positive results that may arise by use of RDTs, the target sample size was inflated by 20% to approximately 120 samples per facility.

### Study population

All patients age ≥6 months presenting to the outpatient department who were diagnosed with malaria by RDT (Malaria Ag Pf/Pan, SD Bioline^®^) or blood smear and no self-reported history of recent exposure to SP or trimethoprim-sulfamethoxazole (within the past 2 months if not pregnant, or during the pregnancy, if pregnant) were consented. In each health facility, consenting eligible patients were consecutively enrolled until the required number of DBS samples (120) were obtained.

### Sample collection procedures

**Fig. 2:**
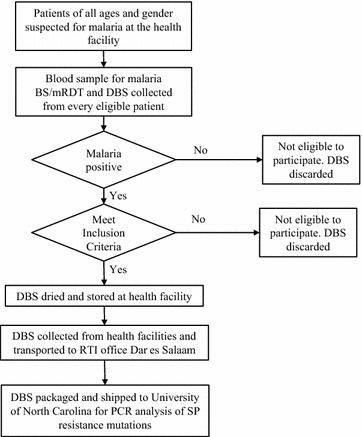
Flowchart for recruiting participants and collecting samples. *BS* blood smear, *DBS* dried blood spot, *RDT* malaria rapid diagnostic test

Two blood spots were collected on filter paper (Whatman no. 3, GE Healthcare Life Sciences) at the time the blood sample was collected for malaria testing; if the RDT was negative or the patient did not consent or otherwise did not meet inclusion criteria, the filter paper was destroyed (Fig. 2). Blood spots from consenting individuals were air dried for 3–4 h on a flat, nonabsorbent surface, then sealed in plastic bags with a desiccant, and stored at room temperature at the study health facility. The samples from all 14 health facilities were packaged in a water-proof container, and shipped to the reference laboratory at the University of North Carolina (UNC) for pooled deep sequencing analyses at three genetic loci associated with sulfadoxine-pyrimethamine (SP) resistance, *dhfr*164, *dhps*540, and *dhps*581 [8, 12–14].

### Molecular methods

#### Optimizing DNA extraction

120 Dried Blood Spots (DBS) from the Kharumwa health center were used in a preliminary experiment to determine if DNA from batched DBS could be extracted reliably. Each subject’s DBS was punched 3 times. One of these punches was added to a single well of a 96-well plate. The other punches were pooled so that wells would contain either three or ten punches from individuals. The plate was extracted using the Chelex method using overnight incubation at 4 °C on a shaking platform at 700 RPM (TPM2 shaker, Sarstedt, Nümbrecht, Germany) [15]. After extraction, 3, 9 and 30 ml aliquots of supernatant were removed from the n = 1, n = 3, and n = 10 extracts, respectively, and combined with aliquots from similarly extracted samples: 1×: aliquots of all 120 individually extracted samples; 3×: aliquots of all 40 of the 3-punch extracts; and 10× aliquots of all 12 of the 10-punch extracts.

Nested PCR amplification was done on each of the three DNA pools for *dhps* and *dhfr* using primers shown in Table 1 based on a previous report [16]. PCR conditions were 95 °C × 2 min, followed by 40 rounds of [95 °C × 30 s → 45 °C × 30 s → 72 °C × 1 min] and a final round of 72 °C for 10 m. Individual indexed libraries for each gene were made for each DBS pooling condition using the NEB Next Ultra DNA Library Prep Kit (New England Biolabs, Ipswich, USA) and pooled in equimolar fashion. Pooled libraries were sequenced on an Illumina MiSeq using 2 × 300 bp reads at the UNC High Throughput Sequencing Facility. Allele frequencies for single nucleotide polymorphisms (SNPs) within the demultiplexed libraries were determined using methods previously described [17, 18].

**Table 1:** Primers

Primer	Sequence	Descrpition
Pfdhfr-F1	TCCTTTTTATGATGGAACAAG	*dhfr* outer forward
Pfdhfr-R1	AGTATATACATCGCTAACAGA	*dhfr* outer reverse
Pfdhfr1-F	TGAGGTTTTTAATAACTACACATTTAGAGGTCT	*dhfr* inner forward
Pfdhfr1-R	TCGCTAACAGAAATAATTTGATACTCAT	*dhfr* inner reverse
Pfdhps-F1	AACCTAAACGTGCTGTTCAA	*dhps* outer forward
Pfdhps-R1	AATTGTGTGATTTGTCCACAA	*dhps* outer reverse
Pfdhps1-F	TGAAATGATAAATGAAGGTGCTAGTGT	*dhps* inner forward
Pfdhps1-R	GTTGTGTATTTATTACAACATTTTGATCATTC	*dhps* inner reverse

Sequencing reads were aligned using bowtie2 with default settings to the *P. falciparum* 3D7 reference strain and the minor allele frequency (MAF) at all positions across the amplicon was determined using a revised version of our previously described Minor Allele Catcher (MAC) [18]. Bases and reads were excluded from analysis if they had a mapping quality less than or equal to 10, a read length less than or equal to 200 bp, a base quality of less than or equal to 20, a base depth of less than or equal to 5000, if SNPs occurred in the first or last twenty-five bases of a read, or if 30% or fewer of the reads were on the forward or reverse strand.

#### Extraction and analysis of DBS from 13 other health centres

Based on the results of this pilot (Table 2), DNA from all of the remaining samples was extracted in batches of 10 by health centre. The DNA extracts from each of the health centres were then pooled yielding one amplicon pool for each site (13 pools). Library preps and analysis were performed as above. Data from the Kharumwa health centre, 10-punch extract, was re-run a second time to generate the 14-health center dataset.

**Table 2:** Malaria testing and positivity rates at selected health centres

Region	District	Health centre	Tested	Positive	Malaria positivity rate (%)
Lake Zone					
Kagera	Karagwe	Kayanga	331	121	37
Kagera	Muleba	Kaigara	292	120	41
Mara	Musoma	Murangi	219	121	55
Mara	Tarime	Sirari	310	122	39
Geita	Geita	Katoro	238	122	51
Geita	Nyang′hwale	Kharumwa	230	120	52
Mwanza	Misungwi	Misasi HC	187	130	70
Mwanza	Sengerema	Mwangika	332	121	36
Sub-total Lake Zone			*2139*	*977*	*46*
Southern Zone					
Lindi	Lindi Rural	Kitomanga	199	126	63
Lindi	Nachingwea	Marambo	244	129	53
Mtwara	Masasi	Nagaga	222	160	70
Mtwara	Mtwara	Nanguruwe	215	138	64
Ruvuma	Songea	Madaba	261	120	46
Ruvuma	Namtumbo	Namtumbo	295	120	41
Sub-total Southern Zone			*1436*	*793*	*55*
Total			*3575*	*1770*	*50*

## Results

A total of 3575 febrile patients were screened, and 1770 were found to be positive for malaria, yielding an overall positivity rate of 49.5% (Table 2). In this pilot work to determine the extraction efficiency of individual versus pooled extraction using the 120 participants from Kharumwa health centre, the proportion of mutant alleles varied only slightly as a result of batching DBS prior to extraction, with 9.4–10.7% alleles mutated at *dhps540*, 2.8–3.9% at *dhps581*, and none at *dhfr164* (Table 3). As these differences in allele frequencies were considered negligible, DBS from all other health centers were extracted in pools of 10.

**Table 3:** Distribution of mutants using pools from individually extracted (1×), 3-punch extracted (3×) and 10-punch extracted (10×)

DNA pool	Proportion of mutant alleles		
	Dhps540	Dhps581	Dhfr164
1×	88.5% (339/383)	2.8% (13/464)	0% (0/319)
3×	88.6% (1014/1145)	3.8% (52/1380)	0.3% (2/586)
10×	89.9% (563/626)	3.3% (25/765)	0% (0/401)

A total of 1750 dried blood spot (DBS) samples were collected (117–160 samples per facility) and used in the final study analysis. Deep sequencing resulted in an average of 341,420 reads at *dhfr* (range 208,769–644,927) and 168,260 reads at *dhps* (range 49,419–313,645). The library from Kharumwa was re-sequenced during this project. Based upon this repeat sequencing (Table 4) and the 3 sequences in Table 3, the allele frequencies ±standard deviations for *dhps*540 and *dhps*581 mutants were 90.0 ± 2.1% and 3.2 ± 0.7%, respectively. The *dhps*540 mutation was common across all 14 sites, with allele frequency ranging from 55 to 98.4%, with higher allele frequency at sites in Lake Zone compared to Southern Zone (Table 4). Frequency of the *dhps*581 mutation ranged from 0 to 2.4%, with the exception of Kayanga health centre (Kagera Region, Lake Zone) where 24.9% of sequences were mutated (Fig. 1). The *dhfr*164 mutation was detected only at Kanyanga health centre (0.6%).

**Table 4:** Summary of drug resistance allele frequencies

Region	District	Health centre	Samples (N)	Mutant, % (number of mutant/total sequences)		
				dhps540	dhps581	dhfr164
Lake Zone						
Kagera	Karagwe	Kayanga	120	89% (154,320/173,457)	24.9% (46,056/185,117)	0.6% (3972/623,835)
Kagera	Muleba	Kaigara	120	91.2% (270,373/296,323)	1% (31,77/313,669)	0% (123/286,155)
Mara	Musoma	Murangi	117	86% (160,431/186,561)	0% (24/196,781)	0% (20/327,806)
Mara	Tarime	Sirari	120	98.4% (262,461/266,632)	0% (71/285,418)	0% (17/249,213)
Geita	Geita	Katoro	122	97.2% (254,986/262,202)	0.2% (547/280,267)	0% 19/310,706)
Geita	Nyang’hwale	Kharumwa	120	92.9% (176,055/189,500)	2.4% (4860/201,156)	0% (23/322,015)
Mwanza	Misungwi	Misasi	130	92.7% (168,879/182,105)	2% (3773/193,348)	0% (15/348,488)
Mwanza	Sengerema	Mwangika	121	93.2% (177,559/190,591)	0.3% (692/202,087)	0% (24/386,750)
Mean allele frequency Lake Zone*			970	82.6%	3.9%	0.1%
Southern Zone						
Lindi	Lindi Rural	Kitomanga	124	94.4% (162,087/171,760)	0% (47/177,058)	0% (11/225,710)
Lindi	Nachingwea	Marambo	129	77.3% (35,351/45,753)	0.1% (57/50,926)	0% (41/231,375)
Mtwara	Masasi	Nagaga	160	68.1% (107,370/157,670)	0% (17/164,456)	0% (46/247,292)
Mtwara	Mtwara	Nanguruwe	129	73.2% (104,952/143,305)	0.8% (1180/154,626)	0% (17/385,082)
Ruvuma	Songea	Madaba	120	92% (71,494/77,751)	0% (11/83,706)	0% (13/324,890)
Ruvuma	Namtumbo	Namtumbo	118	55.7% (26,134/46,901)	0% (12/52,484)	0% (22/318,552)
Mean allele frequency Southern Zone*			780	76.8%	0.2%	0%

* Mean allele frequency for each zone was calculated by averaging the percentage of mutants from each health center

## Discussion

In a survey of parasites from patients at 14 health facilities in the Lake and Southern Zones of Tanzania, the *dhps*K540**E** mutation was very common [in 10 facilities (71%) more than 85% of alleles at *dhps*K540**E** were mutated, and nowhere were fewer than 55% mutated], while the *dhps*A581**G** mutation remained rare and focal, with frequency greater than 2.4% in only one facility, Kayanga Health Facility, where nearly 25% of alleles carried the *dhpsA581G* mutation. The quintuple mutation (represented by the *dhps*K540**E**) has been reported widely across Tanzania, with prevalence ranging from 64 to 98% in one recent report assessing seven regions [10] and 77 to 95% in another [19]. Previous reports from Tanga Region have found a high prevalence of parasites harbouring the *dhps*A581**G** mutation (44% in Korogwe [7], 51% in Muheza [19], and 57% in Bondo [10]). Although the Lake and Southern Zones of Tanzania are not immediately adjacent to Tanga Region, the absence of the *dhps*A581**G** mutation in the majority of sites highlights that there may be considerable geographic micro-heterogeneity. This is supported by data from Kavishe et al., who similarly found the *dhps*A581**G** to be present at high proportion in only Tanga and Kagera Regions, though the prevalence of mutants at their sites were higher than the allele frequencies reported here [10]. This is a reassuring finding, suggesting that IPTp-SP retains efficacy in the majority of Tanzania, but highlighting the need for monitoring in multiple geographic sites.

The pooling and sequencing methodology presented here is a cost-effective alternative to individual allele-specific PCR. Using this 2-step pooling methodology, it was possible to perform 90% fewer DNA extractions and 99% fewer sequencing runs than if samples from each participant had been sequenced individually, saving a substantial amount of both time and money. The individual extraction and bi-directional Sanger sequencing of 1750 samples would cost on the order of $19,000. Here the same data has been compiled for under $4000.

Another advantage of the pooling method is that it allows direct calculation of the allele frequency, rather than prevalence. That is, it calculates the percentage of the parasite population bearing the mutation rather than the prevalence of individuals bearing parasites with mutated alleles. From an evolutionary point of view, the allele frequency of a mutation is more important than prevalence of the mutation because it approximates the likelihood that a mosquito will become infected with a mutant parasite after exposure to a given population.

Allele frequency and prevalence can be different when mixed infections are present and when individuals have infections with varying levels of parasitaemia. Traditionally, prevalence data have to be transformed by a complex equation to yield predicted allele frequencies [20]. However, in general, the allele frequency approximates the prevalence of major strain in a human population, barring any biases [16, 21].

This study has a number of limitations. Samples were collected only from the Lake and Southern Zones, neither of which are immediately adjacent to Tanga region, where the highest prevalence of the *dhps*A581**G** have been reported, thus more studies in those areas are needed to better define the extent of the mutant. With regard to the pooling methodology employed here, while much more cost-effective and time-saving than traditional PCR, it is not possible to trace a parasite strain back to a participant. Also, some haplotypes tend to be amplified better than others, leading to PCR amplification bias. However, newer barcoding methods, such as primer ID [22], compensate for amplification bias and could allow identification of individuals (although DNA would still have to be extracted from individual DBS).

While overall, these data are reassuring with respect to the efficacy of IPTp-SP, there are select areas with a high prevalence of the sextuple mutant, where IPTp-SP may no longer provide a useful benefit against malaria. Spread of this sextuple mutant will threaten the usefulness of SP for IPTp. Given the fact that the quintuple mutant is already found throughout Tanzania, and the sextuple mutant has been found in high prevalence in several sites, continued surveillance in multiple sites, particularly in and around Tanga and Kagera, is warranted to monitor for the spread of the sextuple mutant. The pooling technique presented here provides a highly efficient and cost effective means to screen many samples from multiple sites.

## Conclusion

Although the quintuple mutant was very common at all sampled facilities, *dhps*A581**G** remains geographically restricted, suggesting that IPTp-SP remains effective in most of Tanzania. However, additional surveillance, particularly in and around Tanga and Kagera Region is warranted. This can be achieved efficiently through sequencing pooled samples as described here. Finally, a better understanding of the effect of the *dhps*581 mutant on the efficacy of IPTp-SP is needed.

## Abbreviations

BS: blood slide
DBS: dried blood spot on filter paper
IPTp: intermittent preventive treatment in pregnancy
IRS: indoor residual spray
MFP: malaria focal person
MiP: malaria in pregnancy
OPD: outpatient department
RDT: malaria rapid diagnostic test
SP: sulfadoxine-pyrimethamine
WHO: World Health Organization
